# Can Micro- and Nanoplastics Modify Food-Allergy-Relevant Pathways?—A Comprehensive Narrative Review

**DOI:** 10.3390/ijms27187997

**Published:** 2026-09-08

**Authors:** Natalia Rutkowska, Dawid Wisniewski, Patrycja Rogala, Michal Ostrowski, Sylwia Smolinska-Wilczynska

**Affiliations:** 1 Academic Initiative for Immunology Development, Department of Clinical Immunology, Faculty of Medicine, Wroclaw Medical University, 51-616 Wroclaw, Poland; natalia.rutkowska@student.umw.edu.pl (N.R.); dawid.wisniewski@student.umw.edu.pl (D.W.); patrycja.rogala@student.umw.edu.pl (P.R.); michal.ostrowski@student.umw.edu.pl (M.O.); 2 Department of Clinical Immunology, Faculty of Medicine, Wroclaw Medical University, 51-616 Wroclaw, Poland

**Keywords:** microplastic, nanoplastic, food allergy, intestinal barrier, protein corona, oral tolerance, pediatric exposure

## Abstract

Micro- and nanoplastics (MNPs) are widely detected in food, drinking water, food-contact materials, and human biological samples. This narrative review evaluates whether experimental evidence supports a role for MNPs as modifiers of food-allergen digestion, intestinal-barrier function, microbiota composition, and immune tolerance. In vitro studies indicate that protein-corona formation can alter allergen conformation, epitope accessibility, and proteolysis, although effects vary by polymer, particle size, dose, and digestive model. Rodent studies provide evidence that MNP exposure can disrupt epithelial integrity, induce oxidative and inflammatory signaling, and modify microbiota-dependent immune regulation. More direct food-allergy models have reported exacerbation of ovalbumin- and cow’s-milk-allergic responses, including Th2 polarization and changes in dendritic cell and regulatory T-cell compartments. Infants may represent a susceptible and highly exposed population because of immature digestive and barrier function and the use of plastic feeding equipment; however, the available pediatric evidence is limited to exposure studies and simulated digestion. The effect of MNP exposure on the incidence of food allergies, reaction thresholds, or clinical severity in humans has not yet been studied. Accordingly, current findings support biological plausibility and identify research priorities, but they do not establish causality in humans.

## 1. Introduction

Microplastics and nanoplastics (MNPs) are widespread environmental contaminants generated during manufacture, use, weathering, and degradation of plastic materials. Microplastics are commonly defined as plastic particles smaller than 5 mm, whereas nanoplastics occupy the submicrometer range, although operational definitions vary across studies. Oral exposure occurs through food, drinking water, and food-contact materials, and inhaled particles cleared from the respiratory tract may also be swallowed. Reported exposure estimates vary substantially with sampling strategy, analytical method, polymer, and lower particle-size limit [1,2].

Experimental studies indicate that MNPs can interact with proteolytic enzymes and dietary proteins in the gastrointestinal lumen. Incomplete proteolysis is relevant to allergenicity because resistance to digestion is a characteristic of several food allergens, although it is neither necessary nor sufficient for clinical sensitization [3]. Studies involving β-lactoglobulin, a major cow’s-milk allergen, show that selected polymers can adsorb the protein, alter its conformation, and modify its digestibility and IgE-binding properties in vitro [4]. The direction and magnitude of these effects depend on polymer type, particle size, concentration, surface oxidation, and the digestive model, and do not demonstrate food allergy in humans.

A central mechanism is the formation of a biomolecular corona on the particle surface. Proteins with relatively long residence times form the operationally defined hard corona, whereas more rapidly exchanging proteins form the soft corona [5,6,7]. Corona composition gives a particle a context-dependent biological identity and can alter its interactions with allergens, digestive enzymes, epithelial cells, and immune cells. Particle-bound proteins or protein fragments may be retained from proteolysis for longer than their unbound counterparts, but the extent to which such complexes persist or reach mucosal immune tissue in vivo remains unknown.

Beyond luminal interactions, selected MNP exposures have been associated with epithelial oxidative stress, altered tight-junction proteins, inflammatory signaling, and microbiota perturbation in intestinal cell and animal models [8,9,10,11,12,13,14,15,16]. Barrier disruption could increase contact between incompletely digested antigens and mucosal immune cells. Experimental studies have also reported Th2-associated cytokines and increased IgE in allergy models [17,18,19]. These observations support a mechanistic sequence, but they do not establish that typical human dietary exposure causes a clinically relevant condition previously referred to as “leaky gut” or initiates food allergy.

The intestinal epithelium may connect particle-associated injury with allergic inflammation. Damaged epithelial cells can release IL-25, IL-33, and thymic stromal lymphopoietin (TSLP), which activate group 2 innate lymphoid cells (ILC2s), promote Th2 differentiation, and recruit eosinophils, basophils, and alternatively activated macrophages [20]. Although this alarmin network is well established in type 2 disease, evidence that oral MNP exposure activates the complete alarmin–ILC2 cascade specifically in human food allergy remains indirect.

**Figure 1:**
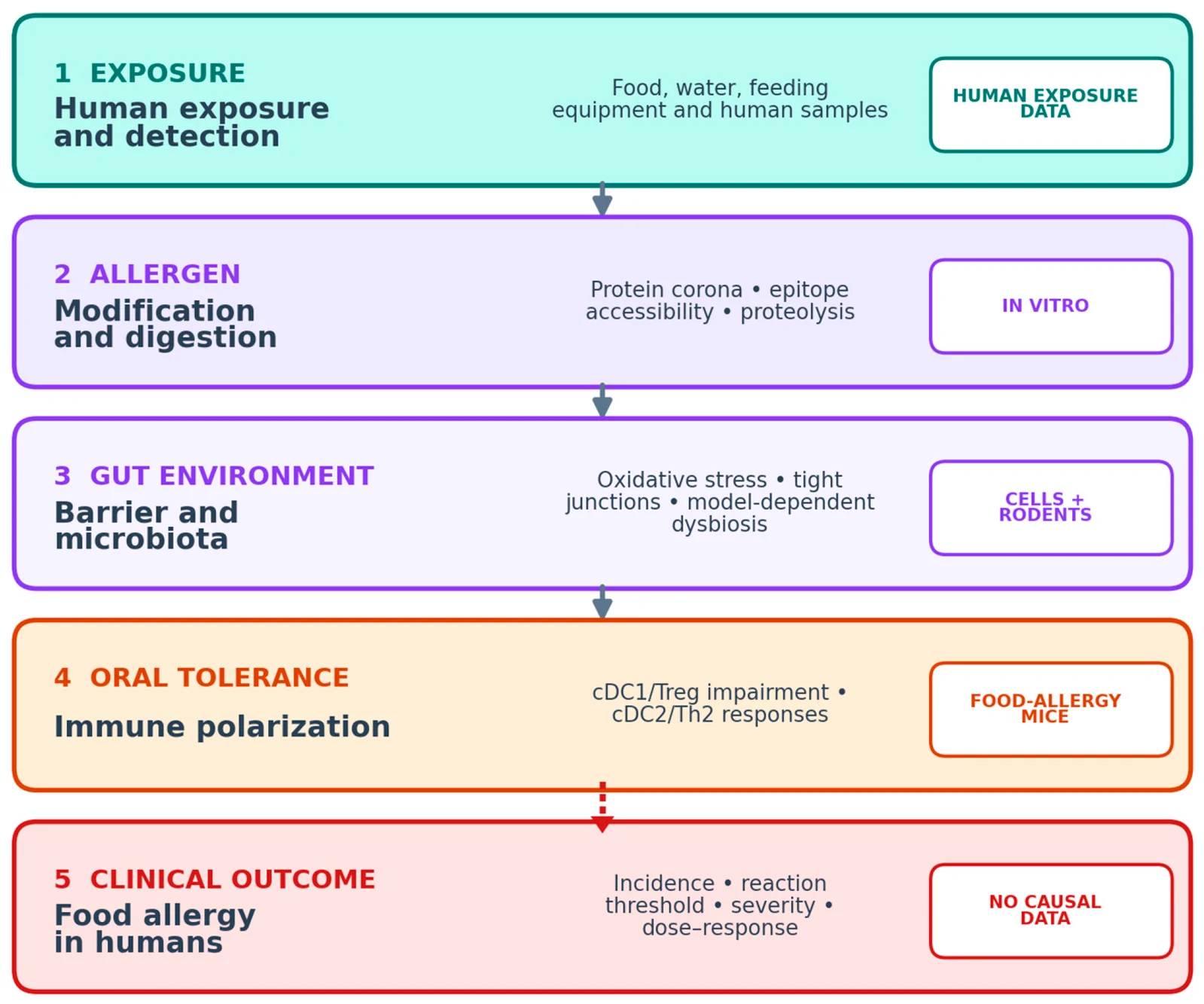
Evidence-supported pathway linking human MNP exposure with experimental mechanisms relevant to food allergy. Solid arrows denote links supported in in vitro or animal models; the dashed arrow marks the unproven translation from experimental immune effects to clinical food allergy in humans.

Together, these observations argue against treating MNPs as biologically inert companions of dietary allergens. Depending on their physicochemical properties, particles may alter the form in which antigen is digested and presented while simultaneously changing the epithelial and microbial environment in which immune recognition occurs. This convergence is especially relevant during infancy, when gastric proteolysis, mucosal-barrier function, microbial colonization, and regulatory immune networks are still developing. At the same time, susceptibility should not be inferred from developmental biology alone: exposure, internal dose, persistence, and a clinically validated allergic outcome must be measured within the same population before pediatric risk can be quantified. The proposed pathway linking human MNP exposure with allergen modification, intestinal-barrier effects, immune polarization, and the unresolved translation to clinical food allergy is summarized in Figure 1.

Previous reviews have raised the possibility that orally ingested MNPs may influence food-allergy-relevant mechanisms, while also emphasizing the limited availability of direct evidence [21]. This review examines direct and supportive evidence for interactions between MNPs and food allergy biology. It distinguishes allergen and digestion studies, intestinal barrier and microbiota mechanisms, food-allergy animal models, and indirect respiratory allergy evidence, with particular attention to pediatric exposure and the absence of causal human clinical data.

## 2. Methods

### 2.1. Search Strategy and Data Sources

A targeted narrative search was conducted in PubMed (U.S. National Library of Medicine, Bethesda, MD, USA), Scopus (Elsevier B.V., Amsterdam, The Netherlands), EMBASE (Elsevier B.V., Amsterdam, The Netherlands), and Google Scholar (Google LLC, Mountain View, CA, USA) to identify publications published between January 2018 and July 2026. All databases were last accessed and searched on 20 July 2026. Earlier seminal studies were retained when required to define protein-corona biology, allergen stability, or established immunological concepts. Reference lists of key articles were also screened. To improve methodological transparency, the review process was informed by the Scale for the Assessment of Narrative Review Articles (SANRA), including explicit search concepts, relevance-based study selection, and clear separation of direct and indirect evidence [22].

The following search concepts and Boolean combinations were used and adapted to the syntax of each database:

Scopus: (microplastic* OR nanoplastic*) AND (“food allergy” OR “food hypersensitivity” OR allerg* OR atopy)

Google Scholar: (microplastic* OR nanoplastic*) AND (“food allergy” OR “food hypersensitivity”)

EMBASE: (microplastic OR nanoplastic) AND (‘food allergy’ OR ‘food hypersensitivity’ OR allerg* OR atopy)

PubMed: (“Microplastics”[Mesh] OR microplastic*[tiab] OR nanoplastic*[tiab]) AND (“Food Hypersensitivity”[Mesh] OR “food allergy”[tiab] OR “food allergies”[tiab] OR allerg*[tiab] OR atopy[tiab] OR atopic[tiab] OR “intestinal barrier”[tiab] OR microbiota[tiab] OR “protein corona”[tiab] OR digest*[tiab]).

### 2.2. Eligibility Criteria

Study types: original in vitro, ex vivo, and in vivo investigations were prioritized. Narrative and systematic reviews were used only to identify mechanisms, terminology, and additional primary studies.

Scope: studies examining MNP interactions with food proteins or digestive enzymes, intestinal epithelial integrity, microbiota, immune tolerance, or experimentally induced food allergies. Respiratory-allergy studies were retained only when they provided clearly labeled indirect mechanistic evidence.

Studies were excluded when they did not evaluate biological effects relevant to the gut–allergen interface, lacked sufficient methodological information, or addressed environmental occurrence without relevance to human exposure or allergy mechanisms. Because this was a narrative review, study selection was relevance-based, and no quantitative meta-analysis was attempted.

Eligibility was assessed independently by five authors, and disagreements were resolved by consensus. Direct food allergy evidence was considered separately from supportive intestinal toxicity evidence and extrapolated respiratory allergy findings.

### 2.3. Quality Assessment and Synthesis of Evidence

Evidence was synthesized into four predefined domains: allergen modification and digestive enzyme interactions; epithelial barrier injury and inflammatory signaling; microbiota and immune tolerance pathways; and direct food allergy outcomes in experimental animals and clinical outcomes in humans. No formal risk-of-bias instrument was applied because of the heterogeneity in models and outcomes. Interpretation therefore emphasized experimental system, particle characteristics, exposure dose, directness to food allergy, consistency across studies, and major limitations.

## 3. Results

### 3.1. Intestinal Barrier Damage and Inflammation

#### 3.1.1. The Role of the Intestinal Barrier in Maintaining Homeostasis

The intestinal barrier is an incredibly important component of the body’s defense system. It separates the internal environment from the external environment, including pathogens, allergens, and toxic substances. It consists of a single-layer intestinal epithelium, whose cells are bound together by tight junctions composed of proteins such as ZO-1, occludin, and claudins, which are responsible for maintaining its integrity and controlling permeability. The intestinal barrier also includes a mucus layer produced by goblet cells, as well as the gut-associated lymphoid tissue (GALT). Proper functioning of the intestinal barrier is essential for maintaining the body’s homeostasis, as it enables the selective transport of nutrients while simultaneously restricting the entry of pathogens and toxic substances into the circulatory system.

Disruption of the intestinal epithelial integrity leads to increased permeability of the intestinal barrier, a phenomenon referred to as “leaky gut”. The consequence of this is the translocation of bacteria, toxins, and allergens into the bloodstream, which can trigger an immune system response and induce chronic inflammation. Currently, dysfunction of the intestinal barrier is considered an important pathogenetic factor in many chronic diseases, primarily inflammatory bowel diseases, metabolic disorders, and food allergies [23].

#### 3.1.2. Exposure Pathways to Microplastics and Their Presence in the Gastrointestinal Tract

Human exposure to MNPs occurs through ingestion of contaminated food and drinking water and through contact with food-processing and packaging materials [2]. Airborne particles cleared from the respiratory tract may also be swallowed. One atmospheric study estimated annual inhalation exposure of approximately 7.37 × 10^4^ particles for children and 1.06 × 10^5^ particles for adults [24], but such estimates are highly method-dependent and should not be equated with gastrointestinal absorption or a biologically effective dose. Environmental occurrence data, including reports of 1000–4000 particles/kg in selected agricultural soils, likewise describe potential sources rather than individual human exposure [10].

The irregular surfaces of weathered MNPs contain pits, grooves, oxidized functional groups, and adsorbed contaminants that can support microbial attachment and biofilm formation. Atmospheric particles have been associated with microbial communities that included Sphingomonas and other taxa capable of colonizing solid surfaces [24]. These observations raise the possibility that MNPs can transport microorganisms or microbial products, but ecological correlations with immune-mediated disease do not establish microbial mediation or allergic causality.

Following oral administration in experimental animals, MNPs have been detected in the gastrointestinal tract and associated with epithelial, inflammatory, microbiota, and metabolic changes [10,11,12]. In mouse studies, polystyrene exposure reduced epithelial cell numbers, increased inflammatory cell infiltration, and altered barrier permeability; dietary fat modified some intestinal and metabolic outcomes [11]. The administered doses and particle preparations differ from typical human exposure, and the extent to which experimental retention represents clinically relevant human bioaccumulation remains uncertain.

The exposure context is therefore inseparable from the biological result. Pristine spherical polystyrene beads used in many laboratory studies differ from irregular, oxidized particles released from packaging or generated by environmental weathering. Food components can promote agglomeration or form a corona before particles reach the intestine, while pH, digestive enzymes, mucus, and bile salts can further modify their surface. Studies that report an administered dose without characterizing these transformations are difficult to compare and may overestimate or underestimate the fraction available to epithelial and immune cells.

#### 3.1.3. The Impact of Microplastics on Intestinal Barrier Integrity

Experimental studies have reported direct effects of selected micro- and nanoplastics on intestinal epithelial cells and mucus production [10,12]. Long-term exposure in mice has been associated with altered villus morphology, reduced colonic mucus layer thickness, ulceration, fibrosis, and, in one model, an approximately 40% reduction in goblet cell number [13,14]. The magnitude of these findings depends on polymer, size, dose, exposure duration, and model and should not be generalized to all MNPs or to human exposure.

Disruption of tight-junction organization is a recurrent experimental finding. Reduced expression of ZO-1, occludin, and selected claudins has been reported in several cell and rodent systems [8,12,13,14,15], and transmission electron microscopy has shown widened intercellular spaces and less closely apposed epithelial junctions in selected models [13,14]. These changes are compatible with increased permeability but do not establish a single primary mechanism or clinically relevant intestinal leakage in humans.

Other studies in groups exposed to microplastics revealed abnormal tight junctions between intestinal epithelial cells, disruption of the nuclear membrane, cell nuclei atrophy, mitochondrial vacuolation, and widening of intercellular spaces [12,14]. A significant drop in the expression of MUC1, associated with mucins essential for the proper functioning of the intestinal barrier, was also demonstrated after exposure to polystyrene microplastics, and the levels of CFTR, NKCC1, and SLC26A6 proteins were significantly reduced as well [12,15,25,26]. These defects lead not only to mechanical dysfunctions of the intestinal barrier but also result in the impairment of its immunological functions [8,13].

#### 3.1.4. Oxidative Stress and Intestinal Epithelial Injury

Oxidative stress is one of the most frequently measured endpoints in experimental MNP toxicity studies. Selected intestinal cell and rodent models report increased reactive oxygen species (ROS) and malondialdehyde (MDA), sometimes with concentration- or time-related responses within the tested range [8,13,15]. These findings indicate oxidative injury under the experimental conditions but cannot be extrapolated directly to human dietary exposure.

Furthermore, in groups of mice subjected to dietary exposure to microplastics, the concentration of superoxide dismutase (SOD) and glutathione peroxidase (GSH-Px)—important enzymes that protect against oxidative stress—was reduced [8,12,14,15].

Studies using polytetrafluoroethylene micro- and nanoplastics in human intestinal cell models reported oxidative stress, mitochondrial alterations, inflammatory signaling, and genotoxic endpoints [13]. These data are specific to the tested particle preparations and concentrations and should not be interpreted as evidence of harm from ordinary cookware use.

Transmission electron and confocal microscopy in selected cell models demonstrated particle internalization and perinuclear or mitochondrial localization [13]. Nanoplastics may be internalized more readily than larger particles because of their size and surface-area-to-volume ratio; however, uptake also depends on agglomeration, surface chemistry, charge, and the biomolecular corona.

Reported mitochondrial endpoints include swelling, changes in membrane potential, release of mitochondrial DNA, and altered mitochondrial biogenesis. Experimental studies also describe changes in Nrf2, NLRP3, ASC, caspase-1 p20, IL-1β, TNF-α, IL-8, endoplasmic reticulum stress genes, and AKT/mTOR-associated autophagy pathways [9,12,13,15]. Oxidative DNA damage was more prominent in smaller particles in some systems, and ROS/METTL3-associated changes were linked with reduced TGF-β1 and VEGF-A and impaired intestinal angiogenic endpoints [15]. These molecular signals describe possible injury pathways rather than a uniform effect shared by all polymers.

Morphological findings provide additional context for these molecular measurements. Selected studies described rounded or shortened mitochondria, reduced expression of genes involved in mitochondrial biogenesis, nuclear membrane abnormalities, nuclear atrophy, and mitochondrial vacuolation after exposure [9,12,13,14,17]. Damage to mitochondrial membranes can increase electron leak and ROS generation, while released mitochondrial DNA can function as a danger-associated signal that promotes inflammasome activation. In parallel, inhibition of AKT/mTOR signaling and induction of autophagy or cell death may reduce epithelial renewal. Because these endpoints were measured across different particles and systems, they should be interpreted as a network of candidate mechanisms rather than a single ordered cascade.

Genotoxicity and impaired tissue repair may further reinforce barrier dysfunction. Oxidative DNA damage increased with dose or exposure duration in some nanoplastic and microplastic models, whereas altered angiogenic signaling was associated with reduced VEGF-A and TGF-β1 and changes in intestinal microvascular endpoints [9,13,15]. The intestine normally depends on rapid epithelial turnover and an intact microvascular supply; simultaneous injury to cellular energy metabolism, DNA integrity, and repair pathways could therefore prolong permeability changes. Direct evidence for this integrated process in humans is not available.

Activation of NF-κB/NLRP3-associated signaling, increased IL-1β or IL-18, and reduced tight-junction proteins have been observed together in experimental cell and animal models [13,14]. This combination provides a plausible connection among oxidative stress, inflammatory signaling, and barrier permeability. Whether comparable intracellular particle burdens and signaling occur at typical human dietary exposure levels is unknown.

#### 3.1.5. Activation of Inflammatory Pathways by Microplastics and Intestinal Epithelial Cell Apoptosis

Studies in animal models have demonstrated an influx of a high number of inflammatory cells, including lymphocytes, into the colonic mucosa and submucosa of mice and rats exposed to microplastics [9,12]. The levels of pro-inflammatory cytokines TNF-α, IL-1β, and IL-6 in the colon of the tested animals increased more than twofold and were progressively higher with increasing concentrations of microplastics to which they were subjected [9]. Similarly, a study showed an increase in the protein expression of pro-inflammatory cytokines TNF-α, IL-6, and IFN-γ, while another study reported an increase in TNF-α, IL-1β, and IL-6, along with a decrease in IL-10 [12,14].

With increasing experimental exposure, studies reported higher mRNA expression of IKKβ, TLR4, and MyD88, increased TLR4 and IKKβ protein levels, and correlations between these markers and TNF-α, IL-6, and IL-1β [9]. Cell viability decreased, and morphology became more rounded at 100 μg/mL, while mouse studies showed activation of NF-κB- and NLRP3-associated proteins, including p65, phosphorylated p65, IKKα/β, IL-1β, IL-18, and caspase-1 [9,14]. These detailed dose-related findings support inflammatory and cytotoxic mechanisms under the tested conditions but do not establish effects at human exposure levels.

Studies performed on animal models demonstrated an increase in the apoptotic rate of colonic cells in groups treated with microplastics. An increase in apoptosis-related proteins was observed: caspase-3, caspase-9, and Bax; simultaneously, the apoptotic rate and protein levels were significantly higher under the influence of 8 μm particles than 70 μm particles. Conversely, the level of the Bcl-2 protein decreased, being lower with 8 μm particles than with 70 μm particles [12].

Together, these comparisons support size-dependent bioactivity within the polypropylene model, but nominal diameter cannot be separated from particle number, total surface area, agglomeration, and cellular uptake. A simple rule that all smaller MNPs are proportionally more hazardous is therefore not justified without matched particle characterization.

### 3.2. Immunological Mechanisms and Intensification of Allergies

Experimental evidence suggests that MNPs may influence immune responses relevant to allergy, although direct evidence is limited to in vitro systems and animal models. The strength of inference depends on whether a study measured a molecular mechanism, immune sensitization, or an actual food-allergy outcome.

Potential effects occur along a continuum from physicochemical modification of an allergen, through epithelial and innate immune signaling, to antigen presentation, T-cell polarization, IgE production, and effector-cell activation. A study that demonstrates one step in this sequence does not necessarily demonstrate sensitization or clinical allergy, but detailed mechanistic findings remain important for defining testable pathways.

**Figure 2:**
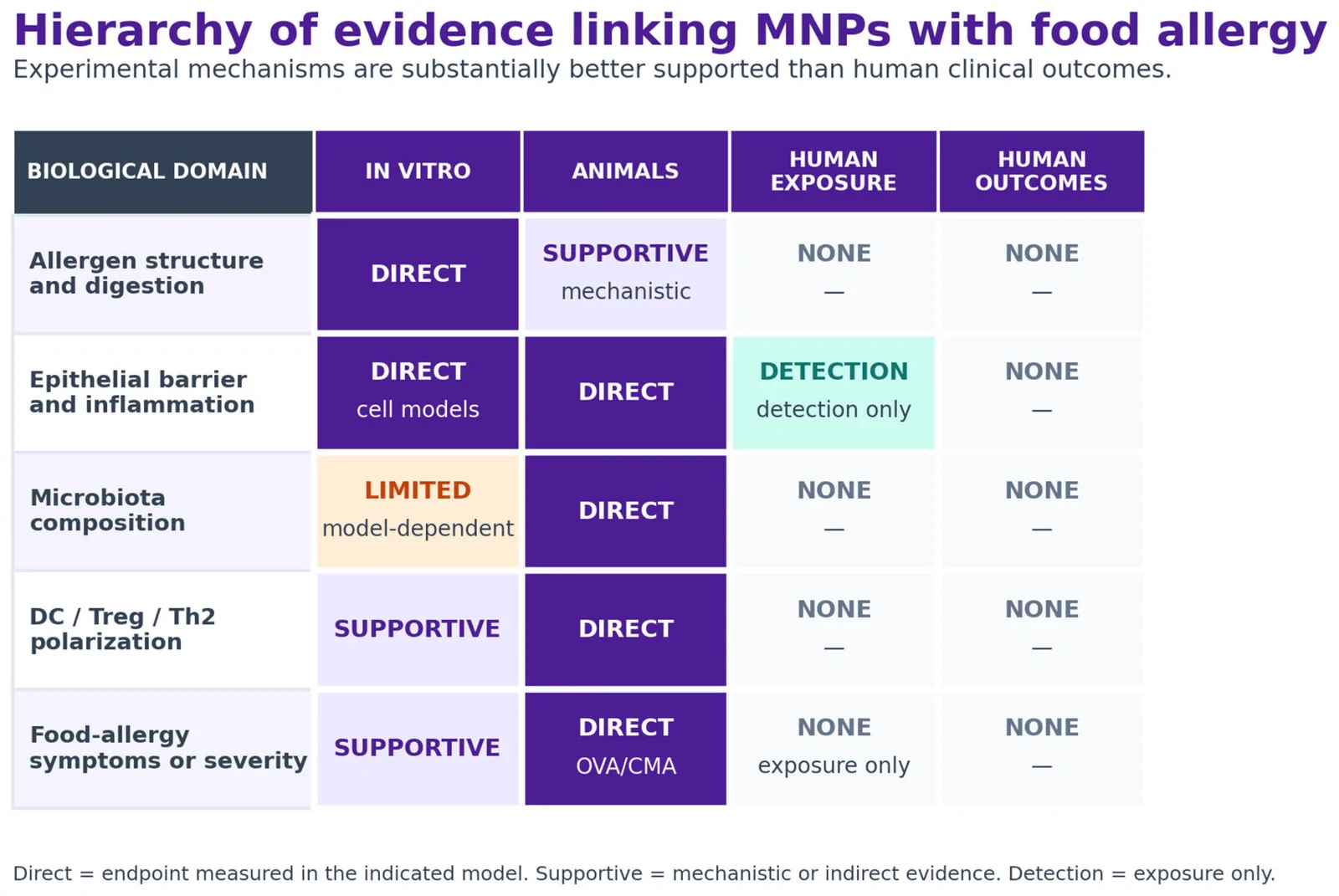
Hierarchy of evidence linking MNPs with food-allergy-relevant outcomes. Direct mechanistic and animal-model evidence is contrasted with human exposure data and the current absence of causal human clinical evidence [4,8,10,11,12,13,14,15,16,18,19,27,28,29,30].

Potential mechanisms include altered allergen digestion, epithelial barrier injury, oxidative stress, microbiota perturbation, and changes in antigen-presenting-cell or T-cell responses. These effects should be described as adjuvant-like or allergy-modifying in experimental models, rather than as proven causes of food allergy. The relative strength and directness of the available evidence across experimental and human settings are summarized in Figure 2.

#### 3.2.1. Alteration of Allergen Structure and Digestibility

MNPs can modify protein digestion through adsorption of allergens and digestive enzymes, formation of biomolecular coronas, and surface-dependent conformational interactions [4,27,28,31]. Weathering and oxidation can increase surface polarity and change which proteins preferentially bind [27,32,33]. The direction and magnitude of these effects are not uniform and depend on the particle, protein, food matrix, and digestive model.

In milk-protein systems, soft- and hard-corona formation can limit enzyme access, retain casein or β-lactoglobulin fragments, and modify the accessibility of known IgE-reactive regions [4,27,28]. These findings establish altered adsorption and digestion kinetics in vitro; their relevance to sensitization, oral tolerance, or clinical reactivity has not been demonstrated in humans.

##### Protein Corona Formation as a Protective Barrier Against Proteolysis

The formation of a corona on the surface of microplastics is a process determined by various parameters: concentration, physicochemical properties of the polymer surface, the polymer surface area available to proteins, digestion kinetics, and the type of substrate. The protein corona is divided into a soft corona with loosely bound proteins and a hard corona where strong binding occurs [28].

The concentration of microplastics directly determines the composition of the corona: in the presence of PS10 microplastics in milk at a concentration of 0.3 mg/mL, a dominance of the hard corona was observed alongside a simultaneous absence of the soft corona [28].

The physicochemical properties of the polymer are of equally crucial importance—Kaseke et al. analyzed the impact of PP-MP oxidation on the digestion of milk proteins and corona formation using an infant digestion model [27]. The study showed that oxidation enriched certain proteins in both coronas. The oxidation process of PP-MP led to a three-fold increase in the binding of whey proteins (14–20 kDa) and enriched the coronas with the lowest molecular weight casein isoforms. Structural changes introduced by additional carbonyl groups and an increased surface polarity of oxidized PP-MP result in stronger binding of certain hydrophilic proteins, which alters the composition of the forming soft and hard coronas [27].

The available particle surface also determines the dynamics of protein binding. In a simulated gastric system, milk-protein fragments associated with 10 μm polystyrene particles, while the pepsin-containing hard corona evolved [28]. Consistent with competitive protein exchange described by the Vroman effect [7], pepsin with higher surface affinity gradually displaced some casein fragments. Particle-associated fragments were digested later than proteins in the bulk solution, with a delay of up to 15 min in that experimental system. This demonstrates transient protection of a fraction of the protein pool, not persistence through the complete gastrointestinal tract.

Digestion kinetics differentiate the durability of both corona types: the soft corona exhibits variability depending on the protein profile of infants and adults—in the latter, no binding of milk proteins was observed [27,28]. The unstable nature of the soft corona stems from weak interactions between proteins, and it can dissociate as a result of prolonged digestion [6]. A similar mechanism was observed by Kaseke et al., where after 5 min certain casein and whey protein bands intensified, and subsequently decreased after 120 min. In the case of the hard corona within the infant digestion simulation, casein bands remained visible even after 120 min [27].

The type of protein substrate is also significant—according to Khatun et al. (2022), caseins are more prone to depositing on the surface of microplastic particles, which is due to their amphiphilic properties and random coil structure, unlike the hydrophilic and compact whey proteins [34,35].

##### Accumulation of Higher-Molecular-Weight Fragments and Implications for Protein Digestion

Simulated digestion studies using polypropylene (PP), polyphenylsulfone (PPSU), polyethersulfone (PES), or 10 μm polystyrene particles reported altered breakdown of β-lactoglobulin, caseins, and other milk proteins [4,27,28]. Cow’s-milk digestion with high concentrations of PS10 produced a transient accumulation of electrophoretic fragments in the 10–35 kDa range and reduced abundance of smaller 2–9 kDa fragments during the gastric phase [28]. These molecular-weight fractions should be distinguished from short peptides of 10–17 amino acids identified by peptidomics [4].

Several parallel processes may contribute: competition between allergens and enzymes for particle surfaces, adsorption-induced conformational change, retention of protein fragments within the corona, and changes in the measurable enzyme pool. One β-lactoglobulin workflow reported decreases of 33% for pepsin, 56% for trypsin, 34% for lipase, and 50% for α-amylase [4]. These values refer to measured enzyme concentrations in that workflow and should not be interpreted as identical reductions in catalytic activity or as proof of impaired nutrient absorption.

Age-related differences in simulated digestion are relevant. Infant models use a higher gastric pH and lower proteolytic capacity than adult models and can therefore magnify particle-associated changes in milk protein breakdown [27,28]. Kaseke et al. reported up to a three-fold slowing of casein degradation under the tested infant-model conditions, whereas adult simulations showed smaller or absent effects [27]. This identifies a mechanistic susceptibility in vitro but does not establish increased food allergy incidence or severity in infants.

The physiological interpretation also depends on the sampling time. A transient increase in larger fragments during the gastric phase may reflect delayed rather than permanently prevented hydrolysis, and later intestinal enzymes may compensate for part of the difference. Conversely, particle-bound material could remain locally concentrated or be delivered farther along the intestine. Current static models do not reproduce gastric emptying, peristalsis, mucus transport, epithelial uptake, or the full sequence of infant gastrointestinal maturation, so neither complete compensation nor persistent antigen delivery can be assumed [36].

##### Modifications of Allergen Structure and Exposure of IgE-Dependent Epitopes

MNP binding can alter the secondary or tertiary structure of food proteins and thereby change the accessibility of existing IgE-reactive regions [4,28]. The available evidence supports altered epitope exposure or persistence rather than the creation of entirely new IgE epitopes.

Spectroscopic and molecular-simulation analyses of β-lactoglobulin showed polymer-dependent changes in secondary and tertiary structure. Selected particles reduced α-helical or β-sheet content and increased exposure of aromatic residues as the protein unfolded; PPSU produced a more polar solvent environment around exposed residues than PP in the tested system [4]. These observations help explain why polymers with different backbones and surface chemistries produced different adsorption and immunoreactivity profiles.

In vitro experiments reported modest but measurable changes in antibody binding. PPSU-associated β-lactoglobulin showed a 5.1% increase in IgG binding and a 3.7% increase in IgE binding, while β-hexosaminidase and histamine release in KU812 cells increased by 9.4% and 65.5%, respectively, under the reported conditions [4]. It is important to emphasize that mast cells and basophils act as the primary effector cells in IgE-mediated immediate-type food allergies. While much of the current in vivo evidence highlights MNP effects on the sensitization phase (involving dendritic cells and T-cell polarization), their potential impact on the effector phase is equally critical. The robust increase in degranulation markers in the KU812 basophil-like cell assay suggests that MNP-allergen complexes might directly enhance the reactivity of these key effector cells, potentially lowering the threshold for allergic reactions [4]. However, in vivo validation of this direct effect on mucosal mast cells is still required. These are molecular and cell-model endpoints and cannot be translated directly into clinical sensitization, mast-cell responses, or reaction severity.

In the β-lactoglobulin study, changes in secondary and tertiary structure varied by polymer. The study also reported altered peptide profiles and increased recognition of known IgE-reactive sequences, illustrating that particle composition can influence the experimental outcome [4].

The biological importance of conformational change depends on whether relevant epitopes remain accessible after gastrointestinal processing and reach immune cells. Neither intestinal transport of the particle–allergen complex nor antigen presentation was measured in the available in vitro digestion studies.

Hard-corona formation can retain protein fragments on particle surfaces and may protect a fraction of bound material from proteolysis [4,28]. This finding should be interpreted as an experimental retention mechanism rather than proof that intact allergens reach gut-associated lymphoid tissue in vivo.

Peptidomic analyses identified differences in the abundance of known IgE-reactive β-lactoglobulin sequences, including regions AA 9–18 and AA 124–135 [4]. The data indicate altered accessibility or persistence of existing epitopes, not de novo formation of allergenic sequences.

Casein-derived fragments were also detected in hard-corona fractions. αS2-casein was prominent, and the AA 171–180 region has previously been associated with persistent cow’s-milk allergy [28,37]. Its presence on a particle does not establish that the epitope remains accessible after intestinal transport or that it elicits an immune response in vivo.

The distinction between structural modification and clinical allergenicity is critical. A change in fluorescence, circular dichroism, peptide abundance, or ELISA binding demonstrates that the molecular presentation of an allergen has changed. Clinical sensitization additionally requires epithelial access, antigen uptake and processing, presentation in an appropriate costimulatory environment, expansion of allergen-specific lymphocytes, and generation of durable IgE responses. The existing β-lactoglobulin experiments address several early and effector-level steps but not this complete sequence.

Particle-associated proteins may remain present during simulated peristaltic and digestive conditions, potentially increasing distal intestinal exposure. However, direct delivery of allergen fragments to gut immune tissue has not yet been demonstrated.

Respiratory-allergen studies, including work with Der p 1, provide indirect evidence that particle–protein interactions can modify allergen recognition [17]. Because exposure route, epithelial environment, and sensitization mechanisms differ, these findings should not be treated as direct evidence for food allergy.

Taken together, the allergen-interaction studies support a testable hypothesis: MNPs may modify the form and persistence of dietary antigens encountered by the mucosal immune system. Confirmation will require integrated in vivo studies that track particles, allergens, antigen presentation, and food-allergy outcomes.

Particle shape and dose add further uncertainty. The baby-bottle-grade PP, PES, and PPSU materials included particles of different sizes and chemical backbones, whereas many mechanistic studies use uniform polystyrene spheres. Adsorption capacity can differ between flakes, fragments, and spheres, and a mass-matched exposure can represent very different particle numbers and total surface areas [4]. Future allergen studies should therefore compare matched particle properties and report both mass and particle-number concentrations.

##### Interactions of Micro- and Nanoplastics with Digestive Enzymes

MNPs can interact with digestive enzymes as well as dietary proteins. Reported effects are polymer-, enzyme-, dose-, and model-dependent; therefore, a generalized statement that MNPs inhibit digestive enzymes is not supported [25,26,27,28,31,38,39,40,41,42].

The high surface-area-to-mass ratio of small particles promotes adsorption of biomolecules and formation of a dynamic protein corona [5]. Corona composition can change the particle’s biological interactions, but the soft- and hard-corona fractions are operational descriptions of exchange kinetics, not fixed multilayer structures.

Tan et al. examined lipid digestion and reported that selected micro- and nanoplastics adsorbed lipase, changed its conformation, and reduced lipolysis in a simulated gastrointestinal system [25]. Zhu et al. likewise found particle-size- and concentration-dependent changes in soybean-oil digestion and ex vivo absorption with polystyrene particles [26]. These studies demonstrate the possibility of enzyme–particle interference but do not establish reduced lipid bioavailability in people.

Pepsin has been studied because it initiates gastric protein digestion. Adsorption to polystyrene was driven partly by π–π interactions between aromatic amino-acid residues and phenyl rings on the particle surface [28]. At a high PS10 concentration, the pepsin-containing corona evolved over time and milk-protein hydrolysis was transiently delayed. Surface chemistry, particle concentration, and gastric conditions are therefore critical determinants of the observed effect.

The next enzyme whose activity has been tested under the influence of MPs is trypsin, the primary protease of the small intestine. As a serine protease, it specifically cleaves peptide chains at the carboxyl end of positively charged amino acid groups, such as lysine and arginine. Previous studies on the impact of polystyrene and polyvinyl chloride on the enzymatic activity of trypsin in marine organisms have yielded mixed results [38,39,40,41,42].

The aforementioned studies also indicated that MPs affect the activity of pancreatic amylase, lipase, chymotrypsin, and carboxypeptidase A, though the findings remain inconclusive [38,40,41,42]. The stimulation of pancreatic enzyme activity by the presence of MPs in the gastrointestinal tract has been attributed to a compensatory secretory response, which is mounted to improve digestion and absorption under challenging conditions [42]. In response to the lack of shared data on mammalian models, Luijic et al. focused their study on the impact of two types of MPs—polypropylene (PP) and polyethylene terephthalate (PET)—on the structure and activity of trypsin, as well as on the in vitro digestibility of beef extract proteins and allergens by trypsin [31].

In the mammalian trypsin system studied by Lujic et al., trypsin and its proteoforms adsorbed more strongly to polypropylene than to polyethylene terephthalate, despite the smaller specific surface area of PP [31]. PET adsorption involved hydrophobic, π–π, and hydrogen-bond interactions, whereas PP binding was dominated by hydrophobic interactions. These differences altered the conformation of the corona-associated enzyme fraction.

PET did not materially alter trypsin tertiary structure, whereas PP caused modest relaxation and minor secondary-structure rearrangement [31]. Overall specific activity in solution remained nearly intact. The low-activity hard-corona fraction represented only about 0.5% of the total trypsin mass, and digestion of the tested beef allergens was not materially impaired even at a high particle concentration.

These findings indicate that trypsin is less affected than pepsin or lipase in the tested systems. In the study by Lujic et al., most trypsin activity remained intact; only the small enzyme fraction associated with the hard corona showed markedly reduced activity [31]. Consequently, enzyme inhibition should not be treated as a universal MNP effect.

##### Implications for Infants

Repeated use and heating can oxidize polypropylene feeding materials and introduce carbonyl-containing surface groups [27,43,44]. Aging changes surface polarity, roughness, and protein affinity, which is important because pristine spherical laboratory particles may not represent particles released from food-contact materials.

In simulated infant digestion, oxidized PP particles bound more than three times as much whey protein as pristine particles and enriched selected low-molecular-weight casein isoforms in corona fractions [27]. The stronger adsorption was associated with delayed proteolysis under the tested conditions. This supports a surface-aging effect in vitro but does not demonstrate infant sensitization or clinical cow’s-milk allergy. Independent static infant-digestion studies without MNPs have shown that the digestion of whey proteins, including α-lactalbumin and β-lactoglobulin, varies with the protein–lactose matrix and digestive phase [45]. Although this study did not evaluate plastic particles, it provides relevant baseline context for interpreting particle-related changes in infant digestion models.

Microplastics were detected in 26 of 34 human breast milk samples in one Raman microscopy study [46]. Among the reported particles, polyethylene accounted for 38%, polyvinyl chloride for 21%, and polypropylene for 17%; detected particles were predominantly 2–12 μm, with 47% in the 4–9 μm range. No association was found with the recorded maternal dietary or personal-care variables. These results document detection in a small cohort but do not establish the maternal transport pathway, infant uptake, co-transport of phthalates, or clinical harm.

The detected particles were described as spherical or irregular, illustrating the diversity that may be missed when exposure is modeled with a single monodisperse polymer. Potential routes into a milk sample include genuine systemic transfer, environmental or procedural contamination, and contact with collection or storage materials; the study design could not fully distinguish among them. Maternal habits captured by questionnaire did not explain detection, but the cohort was too small to exclude dietary, occupational, household, or regional determinants of exposure.

#### 3.2.2. Direct Evidence from Food-Allergy Animal Models

Two recent murine studies provide the most direct evidence relevant to food allergy. In an ovalbumin model, oral nanoplastic exposure increased allergic responses, including allergen-specific IgE, diarrhea, and expansion of intestinal ILC populations; a high-fat diet modified the magnitude of several effects [18]. Because the exposure and dietary conditions were experimental, the findings identify host–exposure interactions rather than a quantitative human risk.

In a cow’s milk allergy model, oral polypropylene exposure aggravated allergic symptoms, specific antibody responses, and intestinal barrier dysfunction and potentiated systemic and local Th2 responses [19]. The study reported depletion of peripheral Tregs and Th2-like Tregs, suppression of tolerogenic cDC1 subsets, expansion of pro-allergic cDC2B cells, increased OX40L expression, and enhanced antigen uptake and Th2 differentiation in a dendritic cell/T-cell co-culture system.

These studies substantially strengthen the biological link between oral MNP exposure, impaired oral tolerance, and food-allergic outcomes in mice. They nevertheless do not establish causality in humans, identify safe or harmful exposure thresholds, or show that infants exposed to them through feeding equipment experience a higher incidence or severity of cow’s-milk allergy.

#### 3.2.3. Epithelial Alarmins and Early Innate Immune Signaling

Epithelial injury can release IL-25, IL-33, and TSLP, canonical early signals that promote type 2 immunity. These alarmins activate ILC2 cells and Th2 lymphocytes and can support recruitment or activation of basophils, eosinophils, and alternatively activated macrophages [20]. Beyond promoting Th2 differentiation, this alarmin network directly influences the immediate-type allergic response. Alarmins released following MNP-induced epithelial injury, particularly IL-33, can prime mucosal mast cells and basophils, significantly lowering their activation threshold for subsequent IgE-mediated degranulation [20]. Although this priming mechanism is well-established in general allergic inflammation, direct in vivo evidence demonstrating that oral MNP exposure exacerbates mast cell or basophil degranulation in clinical food allergy models remains a critical gap in the current literature. Chemokines including TARC/CCL17, CCL22, and CXCL10 have also been discussed in particle-associated immune recruitment, although their roles vary by model.

The temporal sequence is biologically important because epithelial and innate signals can shape the tissue environment before full antigen-specific adaptive responses develop. Nevertheless, the proposition that MNP exposure alone primes the intestine for food sensitization remains a hypothesis unless epithelial injury, alarmin release, ILC2 activation, antigen presentation, and food-allergy outcomes are demonstrated within the same model.

Oral nanoplastic exposure has been associated with expansion of ILC populations in the small-intestinal lamina propria and concurrent microbiota changes in a food-allergy mouse model [18]. Co-occurrence does not identify whether epithelial, microbial, or immune changes are primary, and the direction of microbiota effects differs among studies.

Oxidative stress and NF-κB/NLRP3-associated signaling provide a plausible bridge between epithelial injury, inflammatory cytokine release, and tight-junction disruption [14,47]. These overlapping pathways could amplify one another under sustained experimental exposure, but whether they lower the clinical food-reaction threshold in humans has not been tested.

This network may operate as an amplification loop rather than a linear pathway. ROS can destabilize junctional proteins and promote cytokine release; cytokines can recruit inflammatory cells that generate additional oxidants; and barrier disruption can increase epithelial contact with antigens and microbial products. Alarmins released during injury may then favor type 2 responses in a tissue already exposed to persistent or structurally modified dietary proteins. Demonstrating such a loop will require temporal sampling that shows which event precedes the others and intervention experiments that interrupt individual nodes.

The Okamura food-allergy model is particularly relevant because intestinal ILC changes, microbiota perturbation, and allergic outcomes were assessed in the same animals [18]. Even in that setting, however, the study does not by itself prove that alarmins caused ILC expansion or that ILC expansion mediated the allergic phenotype. Neutralization, depletion, or rescue experiments are required before the alarmin–ILC2 axis can be considered a causal MNP-specific mechanism.

#### 3.2.4. Adjuvant-like Effects, Co-Exposure, and TRPA1 Signaling

Adjuvant-like effects have been observed in experimental models, particularly in respiratory-allergen studies [17]. The Der p 1 data show that polystyrene particles can alter allergen-related responses under defined laboratory conditions, but they do not demonstrate a lower clinical reaction threshold or increased food-allergy risk in exposed people.

The adjuvant concept extends beyond direct particle–allergen binding. Weathered particles can adsorb plasticizers, triclosan, metals, and other environmental chemicals, potentially altering where and how these agents are presented to the intestinal surface. Desorption in food or gastrointestinal fluids, competition with biomolecules, and rapid corona formation determine whether particle-bound transport increases, decreases, or simply redistributes exposure. These parameters were rarely measured together.

Plastic particles can adsorb co-contaminants, including phthalates, in environmental and experimental matrices. Whether particle-bound chemicals meaningfully increase intestinal delivery relative to free chemicals depends on desorption, food matrix, particle properties, and exposure conditions; a clinically relevant vector effect has not been established in humans [48,49,50].

Co-exposure studies indicate that mixtures can produce effects different from those of a single particle or chemical. Associations between childhood phthalate exposure and allergic disease do not establish that MNP-bound phthalates cause food allergy [48].

The TRPA1–p38 MAPK result provides a concrete example of mixture-dependent signaling: combined DEHP and polystyrene exposure enhanced oxidative and Th2-associated responses in an allergic asthma model more than the individual exposures [51]. Triclosan–microplastic combinations have likewise intensified intestinal inflammation and disrupted microbiota–bile-acid pathways in animal studies [50]. The mechanistic value of these experiments lies in showing non-additive mixture effects, not in proving equivalent effects in food allergy or at current human exposure levels.

In food-allergy mouse models, oral MNP exposure has been associated with stronger allergen-specific responses and greater symptom severity, with host diet modifying the effect [18]. These results provide direct animal evidence but cannot be translated quantitatively to human exposure.

#### 3.2.5. Dendritic Cells and Disruption of Oral Tolerance

Intestinal dendritic-cell subsets integrate epithelial, microbial, and dietary signals and help determine whether antigen presentation favors regulatory T-cell differentiation or type 2 sensitization. Under homeostatic conditions, tolerogenic dendritic-cell programs support Treg generation and oral tolerance; danger signals, inflammatory cytokines, or altered microbial metabolites can redirect this balance.

In the weathered-polystyrene study, maturation was reflected in changes in costimulatory markers, including CD80 and CD86, and in altered cytokine production [29]. Weathering is important because oxidation, surface roughness, and adsorbed environmental material can produce a biological identity different from that of pristine particles. The in vitro monocyte-derived dendritic-cell system nevertheless lacks the epithelial, microbial, and dietary signals that normally shape intestinal antigen presentation.

Disruption of tolerogenic dendritic-cell programs is a plausible route to impaired oral tolerance. More direct evidence comes from a 2026 cow’s-milk-allergy mouse model, in which polypropylene microplastics suppressed tolerogenic cDC1 cells, expanded pro-allergic cDC2B cells, reduced peripheral Treg and Th2-like Treg compartments, and enhanced Th2 polarization [19]. Human confirmation is not yet available.

The cow’s milk allergy model connects this general dendritic cell observation with oral tolerance more directly [19]. Suppression of cDC1, expansion of cDC2B, and increased OX40L provide a coherent route from particle exposure to Th2 differentiation, while depletion of peripheral and Th2-like Tregs indicates loss of counter-regulation. Replication with different polymers, doses, and food allergens will be necessary to determine whether the pathway is general or specific to the tested PP–cow’s-milk system.

#### 3.2.6. Respiratory-Allergy Evidence and the Gut–Lung Hypothesis

Inhaled or airborne MNPs have been associated with altered nasal and lung microbiota, epithelial injury, and Th2-associated responses in mice [17,52]. Particle inhalation may also increase respiratory epithelial permeability under experimental conditions. These findings show that exposure route and mucosal site shape particle effects, but they do not establish body-wide immune priming.

Studies involving Der p 1 and other airborne particulates provide indirect mechanistic support for particle–allergen interactions and barrier-mediated sensitization [17,53]. Respiratory and intestinal tissues differ in exposure, epithelial architecture, antigen processing, and microbiota. Claims that MNPs initiate peanut allergy, cross-sensitization, or new food allergy through a gut–lung axis therefore require dedicated experiments and epidemiological confirmation.

A gut–lung connection remains biologically plausible because mucosal immune systems share circulating lymphocytes, microbial metabolites, and systemic cytokine signals. Plausibility is not equivalence: an airborne particle deposited on bronchial epithelium encounters a different barrier, antigen-presenting cell population, and allergen exposure pattern from a particle embedded in a food matrix. Respiratory results should therefore generate hypotheses for intestinal studies rather than fill gaps in direct food allergy evidence.

#### 3.2.7. Intestinal Microbiota and Immune Regulation

Oral microplastic exposure can alter gut-microbiota composition and microbial metabolic pathways in rodents [10,16]. In one six-week polystyrene study, 12–15 bacterial taxa changed, with decreases reported for Parabacteroides, Prevotella, Turicibacter, Bifidobacterium, Phascolarctobacterium, Lachnospira, Adlercreutzia, Blautia, Dialister, and Veillonella, and increases in Coprococcus and Anaeroplasma [10]. These observations describe a specific model rather than a universal MNP-associated signature.

Other experiments reported different or opposing patterns, including changes in Firmicutes, Bacteroidetes, Verrucomicrobia, α-Proteobacteria, Actinobacteria, Oscillospira, Anaerostipes, Ruminococcus, Bilophila, Plesiomonas, Halomonas, and Acinetobacter [11,16]. Differences in polymer, particle size, dose, diet, sequencing platform, housing, and baseline microbiota probably contribute to this heterogeneity. Individual genera should therefore not be assigned fixed beneficial or harmful roles across studies.

Functional analyses identified changes in microbial pathways involving pyruvate and tyrosine metabolism, fatty acid biosynthesis, amino acid metabolism, bile acid profiles, and bacterial interaction with epithelial cells [10,16,50]. These functions may be more informative than isolated taxonomic shifts, but causal mediation of food-allergy outcomes has not yet been demonstrated.

Microbiota perturbation has co-occurred with barrier dysfunction and expansion of innate immune populations in experimental food-allergy models [18]. Establishing mediation will require germ-free, antibiotic, microbial-transfer, or metabolite-rescue experiments that separate a causal microbiota pathway from parallel responses to particle exposure.

Bifidobacterium breve M-16V reduced selected immune and microbiota disturbances induced by nanopolystyrene in an experimental model [30]. This result identifies a potentially modifiable pathway, but it is hypothesis-generating and does not support probiotic treatment recommendations for MNP-related allergic risk.

A major methodological limitation is that many microbiota studies report relative rather than absolute abundance. A relative increase may reflect loss of another taxon rather than expansion of the organism of interest, and cage effects can be comparable to the exposure effect. Standardized sampling, absolute quantification, metabolomics, and functional validation are needed before taxa can be used as biomarkers of MNP exposure or as therapeutic targets.

#### 3.2.8. Synthesis: MNPs as Potential Modifiers of Food-Allergic Responses

The combined evidence supports a multifactorial model in which MNPs may modify food-allergic responses through protein adsorption and corona formation, altered digestion, epithelial oxidative injury, inflammatory signaling, microbiota perturbation, and changes in dendritic-cell, Treg, ILC2, and Th2 compartments [4,10,11,12,13,14,15,16,18,19,27,28,29,30]. These mechanisms may interact, but the evidence supporting each step differs markedly in directness and experimental maturity.

The model is compatible with the epithelial-barrier hypothesis [54], particularly where particle-associated injury coincides with persistent dietary antigen and type 2 immune signals. However, several components—especially the complete alarmin–ILC2 cascade and adjuvant effects involving respiratory allergens—are extrapolated from non-food-allergy systems. The available evidence therefore supports biological plausibility, not causation in humans.

Particle size, polymer, shape, surface charge, oxidation, agglomeration, food matrix, co-contaminants, dose, and host diet can all modify experimental outcomes. Future studies should report these variables and use analytically verified, environmentally relevant particles. Prospective human cohorts are required to relate measured exposure to sensitization, challenge-confirmed food allergy, reaction threshold, and clinical severity.

## 4. Discussion

### 4.1. Digestive-Enzyme Interactions and Food-Protein Digestibility

The collected studies show that interactions between MNPs and digestive enzymes are heterogeneous and cannot be summarized as universal enzyme inhibition [4,25,26,27,28,31,38,39,40,41,42]. Lujic et al. found that even at 20 mg/mL, overall trypsin activity and digestion of the tested beef allergens, including proteins carrying α-Gal epitopes, were not materially reduced; only approximately 0.5% of trypsin was associated with a low-activity hard-corona fraction [31]. Natural resistance of some allergens and prior gastric hydrolysis may have limited the measurable intestinal effect. The physiologic consequences of an allergen-enriched corona remain uncertain because transport and antigen presentation were not evaluated.

Milk-protein studies produced a different pattern. Under simulated infant conditions, selected PP, PPSU, PES, and PS particles delayed degradation of caseins or β-lactoglobulin and altered both electrophoretic fragments and short-peptide profiles [4,27,28]. Reported decreases of 50% for α-amylase, 33% for pepsin, 56% for trypsin, and 34% for lipase refer to measured enzyme concentrations in one workflow and should not be equated with identical reductions in catalytic activity. Likewise, 10–35 kDa bands and 10–17-amino-acid peptides are distinct analytical populations.

Retention of casein or β-lactoglobulin fragments within a hard corona provides a physical mechanism for delayed proteolysis. Casein fragments below 17 kDa were detected on particle-associated fractions even when analogous bands were absent from the surrounding solution at selected time points [28]. This does not prove that intact allergens bypass gastric digestion, cross the intestinal mucus, or reach antigen-presenting cells in vivo. Integrated particle–allergen tracking is needed to test that sequence.

The apparent contrast between pepsin and trypsin studies is informative rather than contradictory. Pepsin interacted with aromatic polystyrene surfaces during the gastric phase, whereas most trypsin remained active in solution despite adsorption of a small fraction to PP or PET [28,31]. Enzyme structure, polymer chemistry, pH, ionic strength, particle concentration, and the presence of competing food proteins all affect corona composition. Risk assessment should therefore be based on defined enzyme–polymer–matrix combinations rather than a single generic “digestive-enzyme inhibition” endpoint.

### 4.2. Allergen Structure Modifications and Exposure of IgE-Dependent Epitopes

Food allergens contain linear epitopes determined by primary sequence and conformational epitopes dependent on protein folding [37]. MNP interaction with β-lactoglobulin altered conformation and the accessibility or persistence of known IgE-reactive sequences, including AA 9–18 and AA 124–135 [4]. The data support exposure of existing regions rather than formation of new epitopes. αS2-casein fragments containing AA 171–180 were identified in corona fractions, but their in vivo immunological relevance was not tested [28,37].

Polymer-dependent effects were quantitatively modest for antibody binding but larger for selected cell degranulation markers. PPSU-associated β-lactoglobulin increased IgE binding by 3.7%, β-hexosaminidase release by 9.4%, and histamine release by 65.5% in a KU812 basophil-like cell assay [4]. These values should not be interpreted as equivalent changes in clinical mast-cell degranulation, reaction threshold, or severity.

Infant simulated digestion differs from adult models through higher gastric pH, lower pepsin concentration and activity, and shorter or developmentally distinct digestive conditions. One model used a gastric pH of approximately 5.0 and pepsin activity of 268 U/mL and reported up to a threefold slowing of casein breakdown in the presence of PP particles [27]. These conditions identify a plausible susceptibility mechanism, but no human infant study has shown increased food allergy incidence or severity.

**Figure 3:**
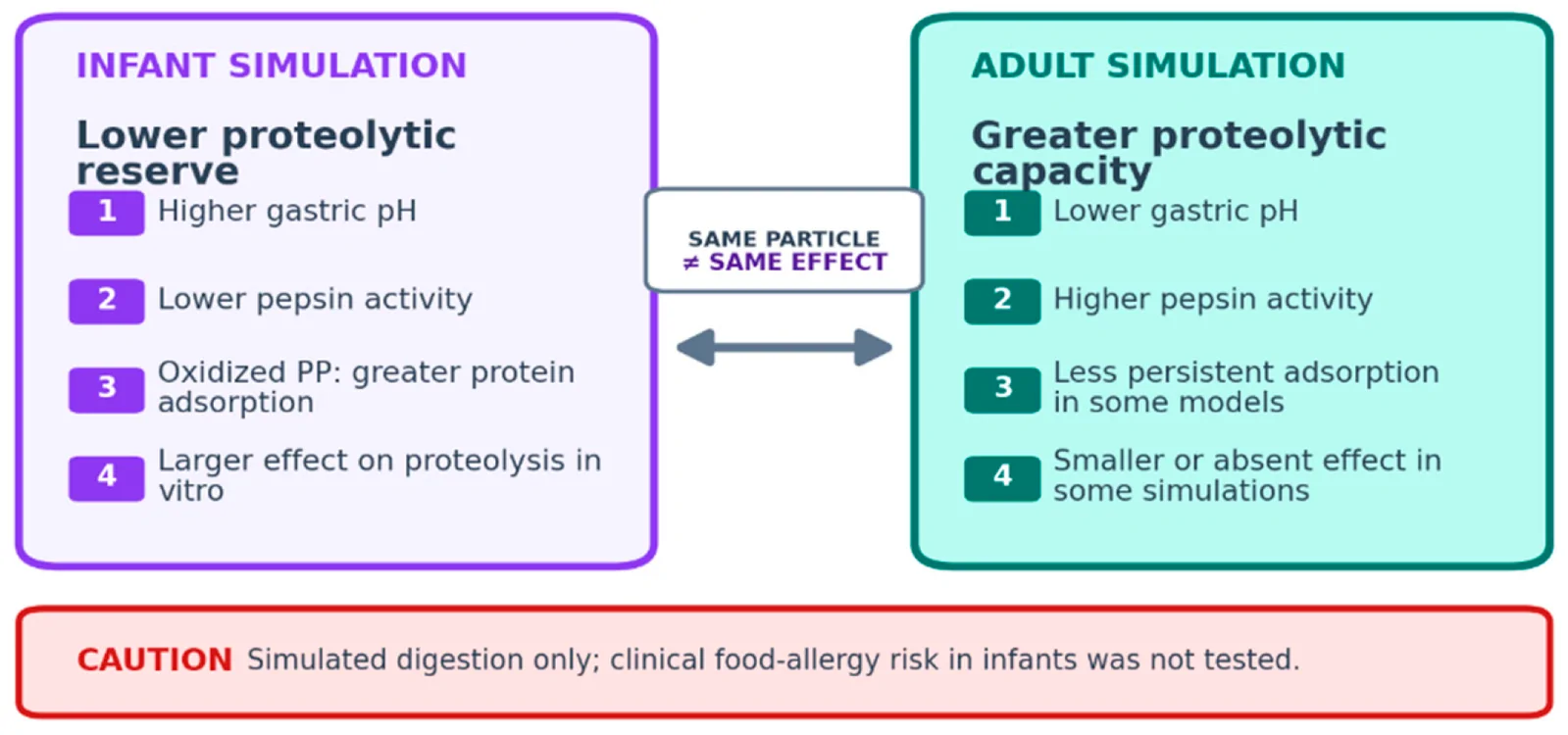
Comparison of infant and adult simulated gastric digestion in studies of MNP–milk-protein interactions. Infant-model conditions may magnify experimental effects, but no clinical food-allergy outcome was measured [27,28].

The β-lactoglobulin data illustrate why multiple analytical layers are needed. Spectroscopy documents protein unfolding, electrophoresis and peptidomics show altered digestion products, ELISA measures antibody binding, and KU812 cells provide an effector-cell readout [4]. Convergence across these assays strengthens the conclusion that selected polymers modify molecular allergenicity in vitro. It still leaves unanswered whether the same complex forms in formula, survives the infant gastrointestinal tract, and changes antigen-specific immunity. The principal differences between infant and adult simulated gastric digestion and their implications for interpreting MNP–milk-protein interactions are summarized in Figure 3.

### 4.3. Plastic Aging and the Particular Susceptibility of Infants

Repeated heating and use can age polypropylene feeding materials. A widely cited experiment estimated release of up to 16.2 million microplastic particles per liter under standardized infant-formula preparation conditions [55]. Oxidation introduces carbonyl-containing groups, increases surface polarity, and can strengthen adsorption of selected milk proteins in simulated digestion [27,43,44]. Release estimates depend strongly on preparation protocol, particle-size detection limits, and analytical method and should not be interpreted as a toxicological threshold.

Microplastics were detected in 76% of breast-milk samples in a study of 34 women, with PE, PVC, and PP among the most frequently reported polymers and most detected particles in the 2–12 μm range [46]. The study’s small cohort, limited sampled mass, and analytical constraints preclude population-level inference. Detection does not confirm a maternal translocation mechanism, carriage of phthalates by the same particles, infant absorption, or clinical harm.

Infancy remains an important susceptibility window because digestive capacity, epithelial barrier function, microbial colonization, and oral tolerance are still developing, while feeding practices may create repeated contact with plastic materials. Nevertheless, simulated digestion and exposure-detection studies cannot establish risks of allergy, obesity, endocrine disease, or chronic inflammation. Dynamic digestion models, validated exposure assessment, and prospective pediatric cohorts are required.

Exposure mitigation hypotheses should also be tested experimentally rather than assumed. Formula preparation temperature, bottle material, repeated use, mechanical abrasion, cleaning, and sterilization can all change particle release and surface aging. Studies comparing realistic preparation protocols with matched nutritional matrices could identify whether simple changes reduce exposure without compromising microbiological safety. Such work would be more actionable than extrapolating clinical risk directly from particle counts.

### 4.4. Intestinal Barrier Damage and Activation of Inflammatory Pathways

Cell and animal studies identify the intestinal barrier as a potential target of MNP toxicity [8,10,11,12,13,14,15]. Reported effects include reduced mucus or goblet-cell measures, altered villus morphology, down-regulation of ZO-1, occludin, claudins, and MUC1, widened intercellular spaces, and increased permeability. These changes could increase contact between dietary antigens and immune cells, but accumulation in the human intestine and clinically relevant barrier failure have not been demonstrated.

Oxidative stress provides a plausible connecting mechanism. Increased ROS and MDA, reduced SOD or GSH-Px, mitochondrial injury, and NF-κB/NLRP3-associated signaling have been reported together with TNF-α, IL-1β, IL-6, and apoptosis-related changes in caspase-3, caspase-9, Bax, and Bcl-2 [8,9,12,13,14,15]. These endpoints support interconnected injury pathways under the tested conditions but do not establish a single initiating mechanism or comparable effects at typical human dietary exposure.

MNP-associated microbiota changes may further interact with barrier and immune responses, but rodent studies do not show a universal taxonomic pattern [10,16]. The original proposed link between Desulfovibrio, α-synuclein aggregation, and Parkinson’s disease is not directly relevant to food allergy and should not be used to infer an MNP–allergy mechanism. Functional microbial pathways and causal mediation require more focused investigation [56].

Barrier injury is also spatially heterogeneous. Mucus depletion, tight-junction loss, epithelial apoptosis, and microvascular changes may occur in different intestinal segments and at different times. A transient molecular change does not necessarily produce sustained whole-gut permeability, whereas repeated injury in a susceptible host could impair repair and oral tolerance. Segment-specific histology, functional permeability tests, and recovery time courses are therefore needed alongside molecular markers.

### 4.5. Immunological Mechanisms and Adjuvant-like Effects

The strongest direct immune evidence now comes from food-allergy mouse models. Oral exposure to nanoplastics or polypropylene microplastics exacerbated experimental allergic responses to ovalbumin or cow’s milk proteins and was associated with allergen-specific antibodies, barrier dysfunction, Th2-skewed immunity, and changes in dendritic cell, ILC, or Treg compartments [18,19]. Crucially, the potential of MNPs to directly or indirectly lower the activation threshold of mast cells and basophils—the principal effectors of immediate-type allergic reactions—represents an important area for future investigation, as current evidence relies heavily on in vitro cell-line assays [4]. These studies move the field beyond mechanistic analogy but still do not define a validated human risk threshold.

Supportive studies show that MNPs can modify allergen conformation, dendritic cell maturation, and immune responses to co-exposures [4,17,29,49,50,51]. Mixtures involving phthalates or triclosan and environmentally weathered surfaces illustrate the importance of surface chemistry and associated contaminants. TRPA1–p38 signaling, however, was demonstrated in allergic asthma rather than intestinal food allergy, and respiratory findings should remain clearly labeled as indirect [51].

The alarmin–ILC2 pathway and epithelial-barrier hypothesis provide useful integrative frameworks, not yet validated therapeutic targets for MNP-associated food allergy [20,54]. The central translational gap is the absence of prospective human data relating measured MNP exposure to incident food allergy, oral-food-challenge thresholds, or severity. Experimental results should therefore guide hypothesis development and study design rather than immediate clinical recommendations.

The evidence hierarchy also clarifies where confidence is strongest. Direct food-allergy outcomes are limited to two mouse models; allergen structure and digestion are supported by detailed in vitro systems; epithelial and microbiota findings recur across several animal studies but vary in dose and particle properties; and respiratory allergy and mixture studies remain supportive or indirect. Presenting these levels separately preserves the value of mechanistic detail without converting plausibility into human causation. The principal evidence domains, experimental models, and limitations relevant to the proposed MNP–food-allergy relationship are summarized in Table 1.

**Table 1:** Evidence domains relevant to the proposed MNP–food-allergy relationship. The table distinguishes direct food allergy evidence from supportive and indirect findings.

Evidence Domain	Models	Key Studies	Main Observation	Interpretation and Limitation
Allergen structure and digestion	In vitro digestion and cell models	[4,27,28,31]	Protein adsorption, altered conformation, and model-dependent changes in proteolysis.	Mechanistic evidence; experimental doses and protocols vary. No clinical endpoint.
Epithelial barrier and inflammation	Intestinal cells and rodents	[8,10,11,12,13,14,15]	Oxidative stress, tight junction changes and inflammatory signaling.	Consistent experimental theme, but human functional data are lacking.
Food-allergy outcomes	OVA and cow’s-milk allergy mouse models	[18,19]	Increased allergic responses with Th2 polarization and altered DC/Treg balance.	Most direct evidence, but restricted to animal models.
Microbiota	Primarily rodents	[10,16,18,30]	Changes in community composition and microbial metabolic pathways.	Direction of taxonomic change is model-dependent; human relevance is unknown.
Adjuvant-like and co-exposure effects	Cell and respiratory-allergy models	[17,29,49,50,51,52,53]	Enhanced responses with aeroallergens, plasticizers, or other contaminants.	Supportive and indirect for food allergy; should not be treated as clinical proof.
Human evidence	Exposure and detection studies	[46,55]	MNPs have been detected in human samples and released from food-contact plastics.	Detection does not establish sensitization, reaction severity, or causality.

Furthermore, recent studies have confirmed the presence of micro- and nanoplastics in human blood, demonstrating that a fraction of ingested or inhaled particles can cross mucosal barriers and enter systemic circulation. This systemic exposure raises the critical possibility that MNPs might modulate immunity well beyond the local intestinal environment. Once in the bloodstream, circulating particles could interact with immune cells and promote a state of low-grade systemic inflammation. In the context of food allergy, such continuous systemic immune modulation could prime effector cells, alter regulatory networks, and act as an adjuvant, potentially lowering the threshold dose required to trigger an allergic reaction upon subsequent allergen exposure. Although direct clinical confirmation of this threshold-lowering effect in humans is still pending, the systemic circulation of MNPs represents a highly plausible pathway that warrants intensive investigation [57].

### 4.6. Research Priorities and Clinical Interpretation

Future research should define windows of susceptibility across the lifespan, particularly early development when oral tolerance and intestinal microbiota are being established. Host diet and metabolic state modified the response to nanoplastics in a food-allergy mouse model, indicating that exposure should be studied together with nutritional and metabolic context rather than as an isolated variable [18].

Priority experimental studies should use analytically characterized particles relevant to food-contact materials and report number- and mass-based dose, size distribution, shape, polymer, aging, charge, agglomeration, protein corona, and co-contaminants. Dynamic gastrointestinal models and in vivo particle–allergen tracking should determine whether complexes survive digestion, cross mucus, reach antigen-presenting cells, and alter oral tolerance.

Prospective pediatric cohorts should distinguish environmental detection, external exposure, internal dose, and biological uptake. Particle analytics and dietary assessment should be integrated with microbiome and barrier biomarkers, allergen-specific IgE, component-resolved diagnostics, physician-diagnosed allergy, and, where ethically appropriate, oral food-challenge outcomes. Until such data are available, clinicians should not infer individual food-allergy risk from MNP detection alone, and regulatory thresholds cannot be derived from the current heterogeneous in vitro and animal literature.

Clinical studies should also account for established determinants of food allergy, including eczema severity, family history, allergen-introduction practices, diet, antibiotic exposure, socioeconomic context, and other environmental pollutants. Repeated MNP measurements would be preferable to a single sample because exposure and analytical recovery may vary over time. Harmonized protocols and blinded outcome assessment will be essential if modest exposure-related effects are to be distinguished from confounding and measurement error.

## 5. Conclusions

Current evidence supports the biological plausibility that MNPs can modify food-allergy-relevant processes, including allergen digestion, epithelial-barrier integrity, microbiota composition, and immune polarization. The strongest direct evidence is confined to experimental food-allergy models in mice, while human studies primarily document exposure or particle detection.

No study has yet established that MNP exposure causes food allergy in humans or changes clinical reaction thresholds. Future research should prioritize standardized particle characterization, environmentally relevant dosing, prospective pediatric cohorts, and clinically validated food-allergy outcomes. Until then, MNPs should be regarded as potential modifiers of allergenicity and oral tolerance rather than confirmed human food-allergy risk factors.

## Data Availability

Data sharing not applicable—no new data generated.
